# The speed of the hair cell mechanotransducer channel revealed by fluctuation analysis

**DOI:** 10.1085/jgp.202112959

**Published:** 2021-08-19

**Authors:** Maryline Beurg, Jong-Hoon Nam, Robert Fettiplace

**Affiliations:** 1 Department of Neuroscience, University of Wisconsin School of Medicine and Public Health, Madison, WI; 2 Departments of Mechanical Engineering and Biomechanical Engineering, University of Rochester, Rochester, NY

## Abstract

Although mechanoelectrical transducer (MET) channels have been extensively studied, uncertainty persists about their molecular architecture and single-channel conductance. We made electrical measurements from mouse cochlear outer hair cells (OHCs) to reexamine the MET channel conductance comparing two different methods. Analysis of fluctuations in the macroscopic currents showed that the channel conductance in apical OHCs determined from nonstationary noise analysis was about half that of single-channel events recorded after tip link destruction. We hypothesized that this difference reflects a bandwidth limitation in the noise analysis, which we tested by simulations of stochastic fluctuations in modeled channels. Modeling indicated that the unitary conductance depended on the relative values of the channel activation time constant and the applied low-pass filter frequency. The modeling enabled the activation time constant of the channel to be estimated for the first time, yielding a value of only a few microseconds. We found that the channel conductance, assayed with both noise and recording of single-channel events, was reduced by a third in a new deafness mutant, *Tmc1* p.D528N. Our results indicate that noise analysis is likely to underestimate MET channel amplitude, which is better characterized from recordings of single-channel events.

## Teaser

Beurg et al. report that the conductance of cochlear hair cell mechanotransducer channels inferred from single-channel events is larger than using noise analysis, which underestimates size due to filtering of fast openings. The difference leads to a first estimate of the channel activation time constant.

## Introduction

In the first step in auditory transduction, sound-induced motion within the cochlea culminates in opening of mechanoelectrical transducer (MET) channels in the stereociliary (hair) bundle of each hair cell (Hudspeth and Corey, 1977; Ohmori, 1985; Crawford et al., 1989; Fettiplace and Kim, 2014). Despite insight into the likely identity of the channel protein as an isoform of the transmembrane channel-like family (TMC1; Kawashima et al., 2011; Pan et al., 2018), the molecular organization of the channel is still uncertain. The central role of TMC1 has been supported by use of *Tmc1* mutations in mice, which have been demonstrated to alter MET channel properties, including Ca^2+^ permeability (Kim and Fettiplace, 2013; Beurg et al., 2015a; Corns et al., 2016; Beurg et al., 2019) and possibly single-channel conductance (Pan et al., 2013). However, the changes in conductance are disputed (e.g., M412K or *Beethoven* [Pan et al., 2013; Beurg et al., 2015a] and D569N [Pan et al., 2018; Beurg et al., 2019]). Since channel conductance has been used to infer the effects of genetic and chemical manipulations on transduction (Pan et al., 2013, 2018; Effertz et al., 2017; Cunningham et al., 2020), it is important to have a reproducible baseline value from which to judge perturbations. Recording from cell-attached patches on the tips of the stereocilia where the channels are located (Beurg et al., 2009) has not proved a viable option for assaying single MET channel properties, but two other main approaches have been used. One method infers conductance from analysis of current fluctuations (noise; Holton and Hudspeth, 1986; Pan et al., 2018); the other records single-channel events after destruction of most of the tip links, with Ca^2+^ buffers leaving one or two intact (Crawford et al., 1991; Ricci et al., 2003; Beurg et al., 2006; Kim et al., 2013). The channel conductance derived from noise analysis may be underestimated if the channel gating is fast relative to the output filtering of the current (Heinemann and Conti, 1992; Alvarez et al., 2002). The mouse cochlea encodes frequencies up to 70 kHz (Taberner and Liberman, 2005), implying that MET channel gating is likely to be very fast in this animal. This raises concerns about using noise analysis to compare channel conductance values in *Tmc1* mutations to infer channel structure (Pan et al., 2018). We have compared the two methods to assess the error introduced by filtering during noise analysis. Quantification of the error permits an estimate of the channel activation kinetics, which so far have been too fast to measure using conventional stimulation and patch recording techniques.

## Materials and methods

### Mouse mutants

The care and use of animals for all experiments described conformed to National Institutes of Health guidelines and were approved by the institutional animal care and use committee at the University of Wisconsin–Madison. *Tmc1* p.D528N was made by Horizon Sage Labs using CRISPR/Cas9 technology, and the mutations were verified by 500-basepair sequencing around the mutation site (Beurg et al., 2021). Such mice were subsequently bred for five generations, after which any off-target effects should have been eliminated. *Tmc2* knockout mice (B6.129S5-*Tmc2^tm1Lex^*/Mmucd) were obtained from the Mutant Mouse Regional Resource Center, University of California, Davis (Kim and Fettiplace, 2013). All channel properties were studied on a *Tmc2^−/−^* background to avoid complications due to potentially different channel properties of TMC2 (Kim et al., 2013). Neonatal mice were killed by decapitation according to the animal protocol approved by the institutional animal care and use committee at the University of Wisconsin–Madison. For all genotypes, a mixture of male and female mice was used, and no sex-specific effects were noted. Mice were kept on a 12-h light/dark cycle and were allowed solid food and water ad libitum.

### Electrophysiology

MET currents were recorded from outer hair cells (OHCs) and inner hair cells (IHCs) in isolated Organs of Corti of mice between postnatal day 2 (P2) and P7, applying recording and stimulation methods previously documented (Kim et al., 2013; Kim and Fettiplace, 2013). Apical (low-frequency) and basal (high-frequency) turns were ∼70% and 20%, respectively, of the distance along the cochlea from the stapes. The recording chamber was perfused with saline containing 152 mM NaCl, 6 mM KCl, 1.5 mM CaCl_2_, 2 mM Na-pyruvate, 8 mM D-glucose, and 10 mM Na-HEPES, pH 7.4. Patch electrodes were filled with a solution of 130 mM CsCl, 3 mM MgATP, 0.5 mM Na_2_GTP, 10 mM Tris phosphocreatine, 1 mM EGTA, and 10 mM Cs-HEPES, pH 7.2, and connected to an Axopatch 200B amplifier. Electrode series resistances with 60% compensation were ∼3 MΩ, which with a 5-pF cell capacitance gave a recording time constant of 15 μs, equivalent to a bandwidth of 10.6 kHz. Whole-cell currents were low-pass filtered with an 8-pole filter (Frequency Devices), usually with a corner frequency of 10 kHz, comparable to the recording system bandwidth; in some experiments, the corner frequency was reduced to 2.5 kHz. Experiments were performed at room temperature (21°C–23°C). Results are presented as mean ± 1 SD, and the statistical test of significance was a two-tailed *t* test.

### Single-channel analysis

**Figure 1.**
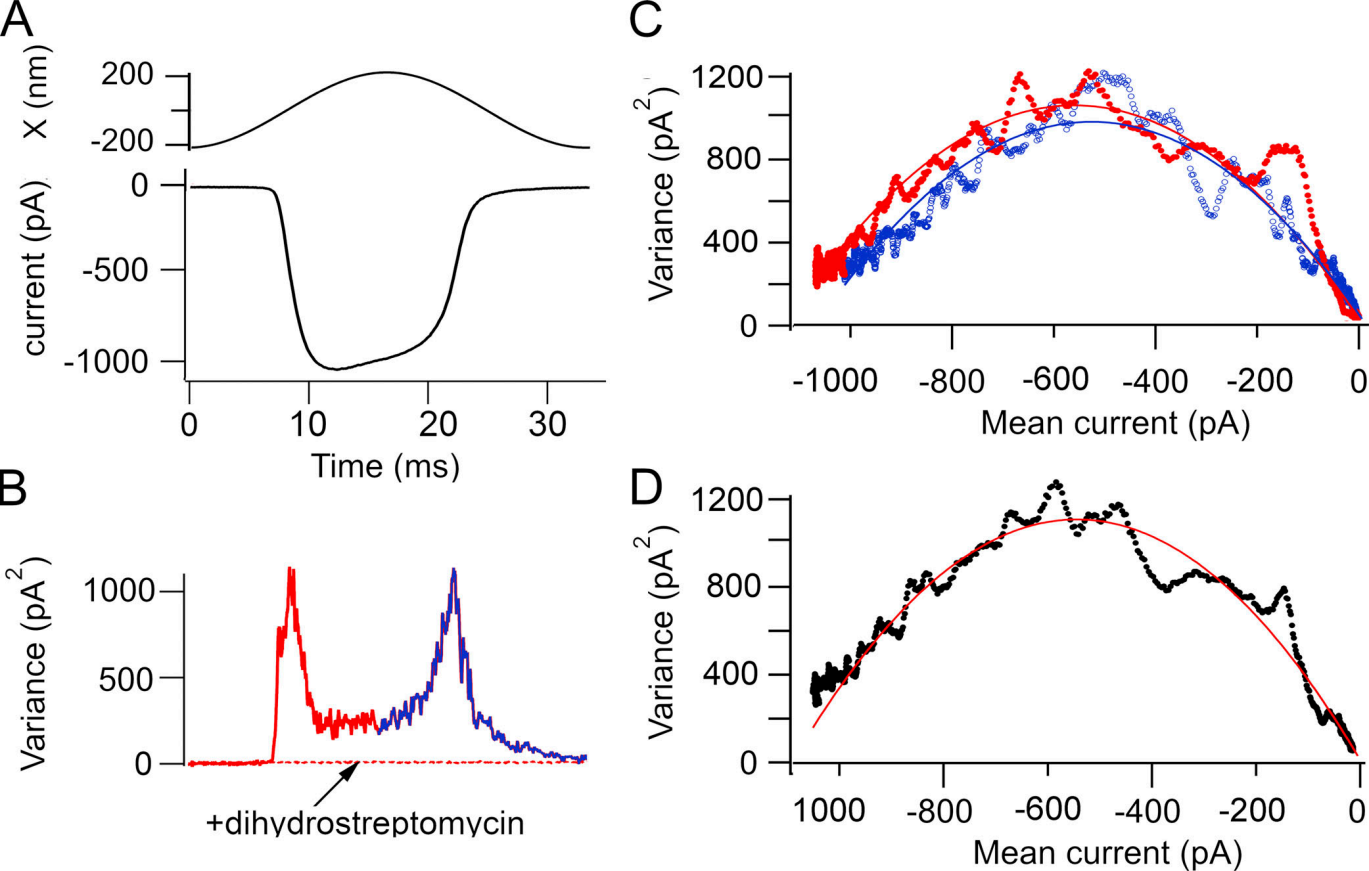
**Method of nonstationary fluctuation (noise) analysis of MET currents in a P4 basal OHC.** **(A)** Mean MET current in response to a 30-Hz sinusoidal fluid jet deflection of the hair bundle. 40 traces averaged. **(B)** Mean variance of the MET current determined by subtracting the mean current from each of the responses, squaring, and averaging (red on the rising phase and blue on the falling phase of the current). The variance is maximal when the channel open probability is ∼0.5. The current variance is abolished by perfusion of 0.2 mM of the MET channel blocker dihydrostreptomycin. **(C)** Plot of the variance against the mean current for the rising phase (red) and the falling phase (blue) of the current. Smooth parabolas are fits with Eq. 1 to give values for the single-channel current (red, i = −3.8 pA; blue, i = −3.4 pA). **(D)** Variance for the rising phase (segment from 0 to 16.7 ms) was averaged with variance of the falling phase (segment from 33.3 to 16.7 ms) and plotted versus mean current. Red line is fit to Eq. 1, with i = −3.7 pA and N_MET_ = 322 channels. Holding potential, −84 mV.

Stereociliary bundles were stimulated with a fluid jet or a glass probe driven by a piezoactuator (Beurg et al., 2006; Johnson et al., 2011). The bundle motion in some experiments was calibrated by projecting the bundle image onto a pair of photodiodes (Crawford and Fettiplace, 1985; Ricci et al., 2000). Single MET channel events were recorded in whole-cell mode after brief exposure to saline containing 5 mM 1,2-bis(o-aminophenoxy) ethane-*N*,*N*,*N*,*N*-tetra-acetic acid (BAPTA) plus 2.5 mM Ca^2+^ (Beurg et al., 2006; Beurg et al., 2018), and currents were low-pass filtered at 5 kHz. The predominant conductance level was reported in preference to a minority subconductance state (Beurg et al., 2018). Histograms of channel amplitudes were fit with two Gaussians using a routine in Igor Pro 8 (Wavemetrics). Single-channel parameters were also derived from nonstationary noise analysis (Neher and Stevens, 1977; Cull-Candy et al., 1988). In this method, macroscopic MET currents were recorded for two cycles of a 30-Hz sinusoidal deflection of the hair bundle with a fluid jet evoking a near-maximal response. Stimuli were delivered at a low rate, once every 2 s, to avoid decline in the MET current amplitude during a sequence of 40–90 presentations. The digitized currents were low-pass filtered at 10 kHz, digitized at 100 kHz (Fig. 1 A), and analyzed by subtracting the mean current I from each of the individual traces and then squaring and averaging the differences to yield the mean current variance σ_I_^2^. The variance was corrected by subtracting that attributable to the background noise, determined by perfusion of 0.2 mM dihydrostreptomycin to block the MET channels (Fig. 1 B). The variance may be overestimated because of factors other than channel fluctuations contributing to the current noise, such as slight variations in the current amplitude or stimulus onsets. We attempted to exclude such noise sources by selecting runs of current responses to ensure as far as possible exact superposition in both current amplitude and onset time course. A plot of σ_Ι_^2^ against I was fit using a routine written in Igor Pro 8 with Eq. 1:

$$\sigma_{I}{}^{2}{= i\cdot I – I}^{2}{\mathrm{/N}}_{\mathrm{MET}},$$ (1)

enabling i, the single channel current, and N_MET_, the number of MET channels, to be determined. Since σ_Ι_^2^ exhibited peaks on both the rising and falling phases of the current (Fig. 1 B), two semicircular plots could be constructed, and the fits gave similar channel current values, e.g., −3.8 pA for the rising phase and −3.4 pA for the falling phase (Fig. 1 C); averaging the rising and falling phases of the current and the variance yielded a unitary current of −3.7 pA (Fig. 1 D). Channel parameters were subsequently inferred from the average of the rising and falling phases.

### Simulation of MET channel gating

Stochastic two-state (closed to open) channel kinetics were simulated as previously described (Nam and Fettiplace, 2008), and the activation (α) and deactivation (β) rates were defined as $\alpha \left(t\right)=A_{0}exp\left(\Delta E\left(t\right)/k_{B}T\right)$ and $\beta \left(t\right)=A_{0}exp\left(-\Delta E\left(t\right)/k_{B}T\right),$ where *A*_0_ is a rate constant, Δ*E* is the mechanical energy of a channel, and *k_B_T* is the thermal energy scale. Use of a two-state rather than the three-state Boltzmann was based on previous fits to current–displacement curves (Fettiplace and Kim, 2014). For each stimulus level, channel activation was varied according to a random number generator. *A*_0_ was set to 30 ms^−1^, resulting in a 10-μs activation time constant at the channel open probability of 0.1. Δ*E* was changed linearly from −6 *k_B_T* to 6 *k_B_T* over a 10-ms time span so that the channel went from largely closed to open during the 10-ms simulation period. Channel adaptation was not routinely incorporated, but evidence suggests that with a time constant of 0.2 ms (Kennedy et al., 2003), it did not affect the analysis. Activation of 120 or 200 channels was repeated 100 times to generate a pool of data for statistical analysis. The output was filtered with an 8-pole low-pass Bessel filter with a corner frequency that was varied between 2.5 and 80 kHz. The source code for the model is available from J.-H. Nam (jnam4@ur.rochester.edu).

## Results

### Two methods for determining channel conductance in OHCs

**Figure 2.**
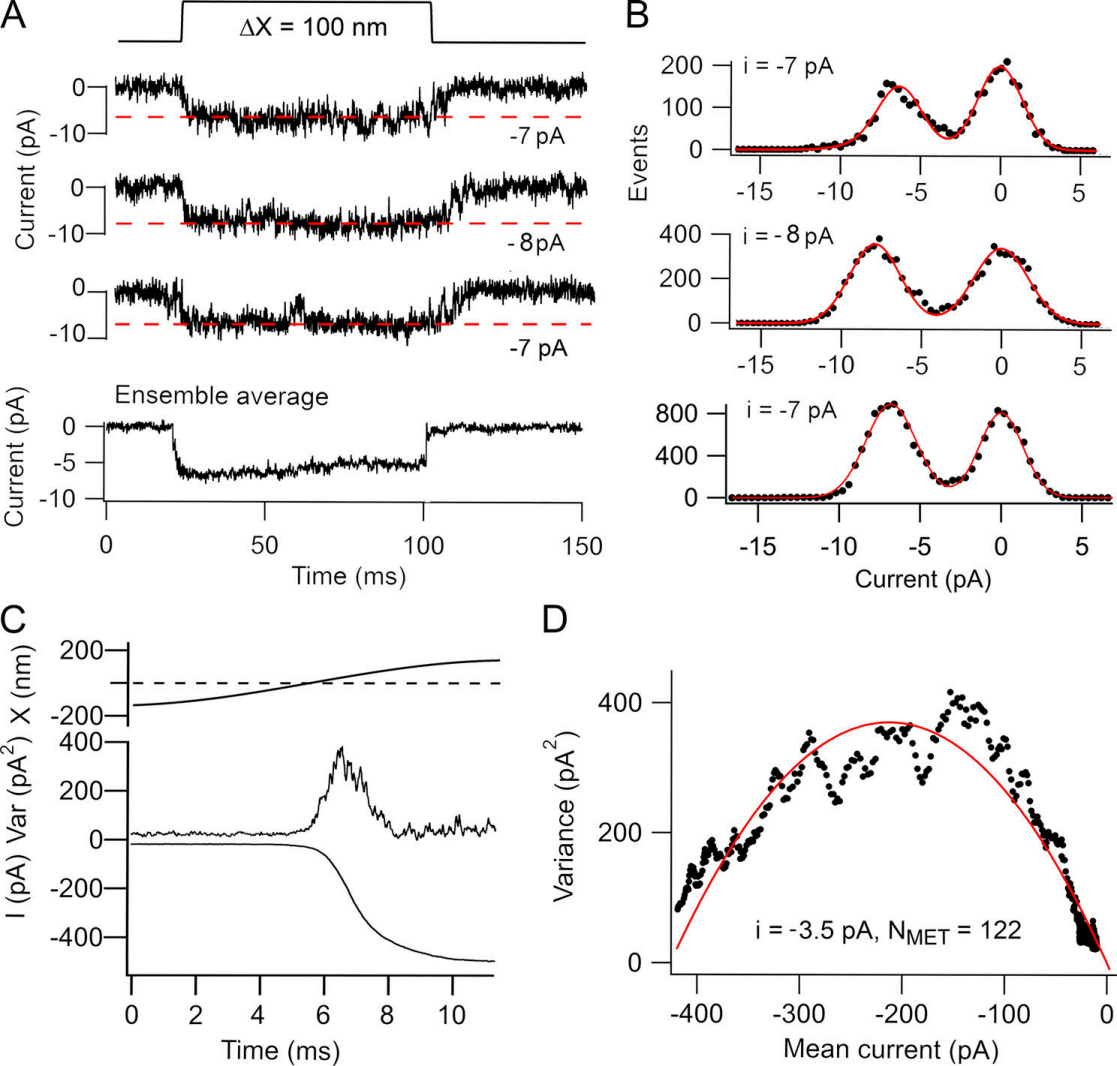
**Comparison of noise analysis and single-channel events in a P4 apical OHC of a *Tmc1^+/+^; Tmc2^−/−^* mouse.** **(A)** Examples of three channel events for bundle deflection with ensemble average of 35 stimuli shown below. Red dashed lines correspond to estimate of channel size in each trace. **(B)** Amplitude histograms of the three recordings in A, with two Gaussian fits with peak currents denoted on each panel. **(C)** Response to a half cycle of sinusoidal bundle deflection showing the mean current (bottom) and the current variance (top). **(D)** Plot of variance (average of the rising and falling phases) against mean of current; smooth red line is fit with Eq. 1 to give i = −3.5 pA and N_MET_ = 122 channels. Noise analysis was performed first, and BAPTA was then applied to obtain single-channel events. Holding potential, −84 mV. Var, variance.

We determined the MET channel conductance using two methods: recordings of single-channel events and analysis of noise in the macroscopic current. The results indicated that the two methods gave different values. For some OHCs, it was possible to apply both techniques to the same cell, the noise analysis being first performed on the macroscopic current and then BAPTA-containing saline perfused to reveal single-channel events. In one such apical OHC (Fig. 2), the single-channel events had amplitudes between −7 pA and −9 pA, with a mean single-channel current of −7.3 ± 1.0 pA (*n* = 46 channel events). For the noise analysis, the variance of the macroscopic current during bundle displacement (Fig. 2 C) was plotted against its current amplitude (Fig. 2 D) and fit with Eq. 1 to yield a single-channel of −3.5 pA. This value, corresponding to a conductance of 42 pS, is about half the size of that from monitoring channel events. Five apical OHCs were characterized following BAPTA treatment, yielding channels with a mean current amplitude of −7.4 ± 0.6 pA and a mean conductance of 85 ± 3 pS (*n* = 5), a value similar to those reported Beurg et al. (2015b, 2021). The mean single-channel currents inferred from noise analysis in apical OHCs was −3.6 ± 0.4 pA (*n* = 10). The ratio of the unitary current inferred from the noise (*n* = 10) and from single-channel events (*n* = 5) was 0.49.

**Figure 3.**
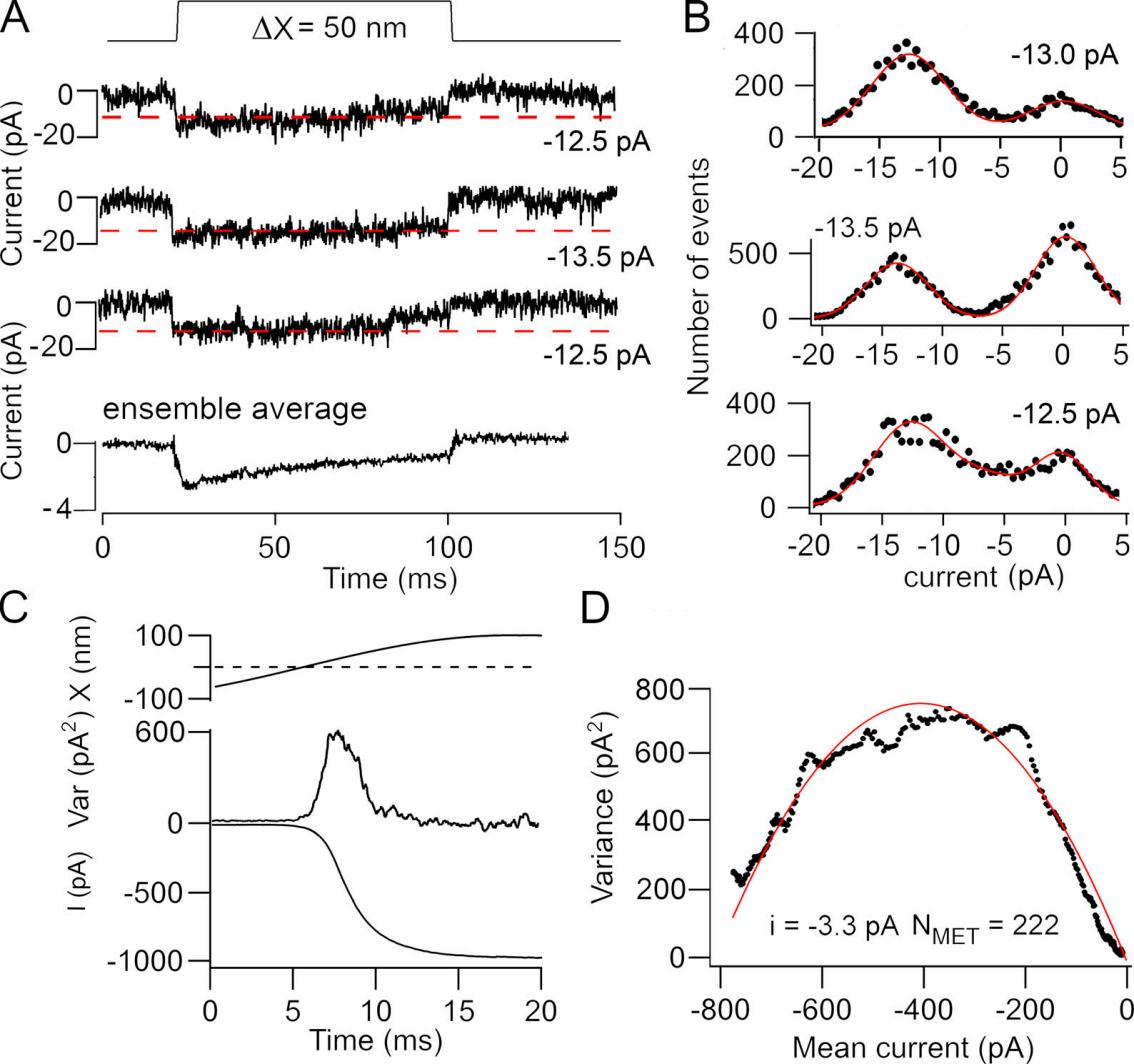
**Comparison of noise analysis and single-channel events in a P3 basal OHC of a *Tmc1^+/+^; Tmc2^−/−^* mouse.** **(A)** Examples of three channel events for bundle deflections and the ensemble average of 50 stimuli shown below. The decline in the ensemble average with time is a manifestation of channel adaptation as previously observed (Ricci et al., 2003). Dashed red lines correspond to best estimate of channel size. **(B)** Amplitude histograms of the three recordings in A, with two Gaussian fits with peak currents denoted on each panel. **(C)** Mean response to a half cycle of a 40-Hz bundle deflection showing the current (bottom) and the current variance (top). **(D)** Plot of variance against mean current fit with Eq. 1 to give single-channel current of −3.3 pA and N_MET_ = 222 channels. Noise analysis was performed first, and BAPTA was then applied to obtain single-channel events. Holding potential, −84 mV. Var, variance.

Both methods for determining channel conductance were also applied to the same basal OHC (Fig. 3). Single-channel events (Fig. 3, A and B) were analyzed from 40 responses to small stimuli, generating discrete current transitions of different mean amplitudes between −9.0 pA and −14.0 pA, and a mean channel current of −11.0 ± 1.1 pA was determined. The decay of the ensemble average is a manifestation of channel adaptation as previously observed (Ricci et al., 2003). In contrast to the single-channel events, noise analysis (Fig. 3, C and D) yielded a single-channel current in the same cell of −3.3 pA flowing through 222 channels. The mean single-channel currents determined from noise analysis was −3.7 ± 0.4 pA (*n* = 8). It was not possible to obtain single-channel events from all these cells, but recordings in four of those basal OHCs gave a mean current of −12.8 ± 1.5 pA (equivalent to a unitary conductance of 152 ± 18 pS), comparable to earlier results (Beurg et al., 2015b, 2018). The ratio of single-channel currents inferred from noise analysis and observations of unitary events in basal OHCs was 0.29, smaller than for apical OHCs.

### Relation between channel number and MET current amplitude

**Figure 4.**
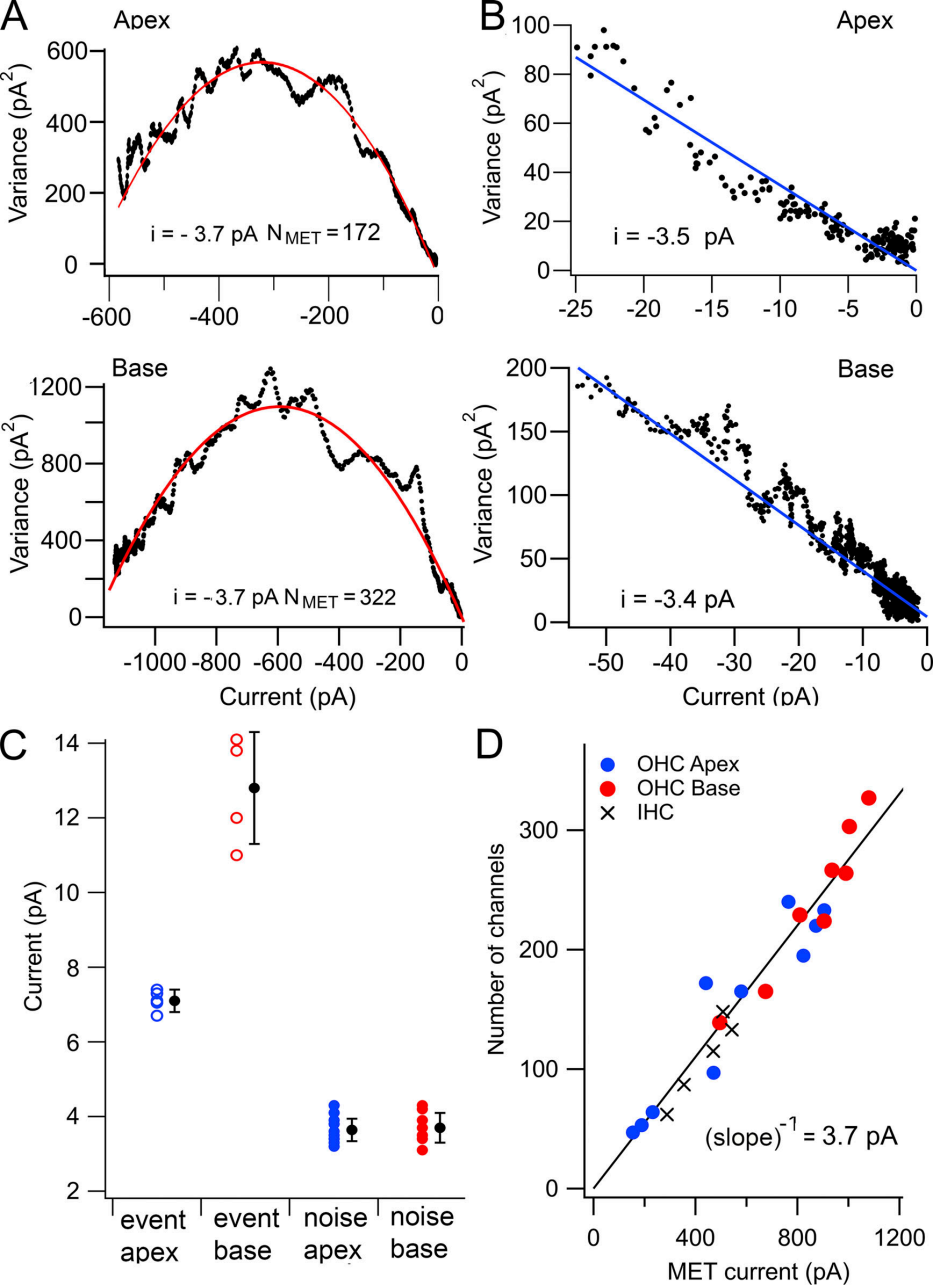
**Comparison of results in apical and basal OHCs. (A)** Variance versus mean current plots for two OHCs from apex (P5, top) and base (P3, bottom); red parabolic line fit to experimental recordings gave similar single-channel currents, i, with different channel numbers, N_MET_. **(B)** Linear approximation of variance versus mean current for small open probabilities (5% of maximum current) from apical (top) and basal OHCs (bottom) fit with straight lines to give i = −3.5 pA (apex) and −3.4 pA (base). **(C)** Collected results of OHC single-channel currents for unitary events after BAPTA treatment for apex (*n* = 5) and base (*n* = 4) and for noise analysis for apex (*n* = 10) and base (*n* = 8). Means ± SD (black circles). **(D)** Number of channels inferred from noise analysis plotted against maximum MET current for apical and basal OHCs fit with a line through the origin of inverse slope of 3.7 ± 0.1 pA. IHC results (crosses), when fit independently, gave single-channel conductance of 3.9 ± 0.2 pS. Holding potential, −84 mV. Collected measurements on apical OHCs and IHCs from P4–P7 mice and on basal OHCs from P3–P4 mice.

Current noise was analyzed in 10 OHCs from apical and 8 OHCs from basal cochlear locations of *Tmc1^+/+^*; *Tmc2^−/−^* mice and used to determine the conductance and number of MET channels. Variance versus mean plots to the noise gave similar values for the channel current in both apical and basal OHCs but with different channel numbers (Fig. 4 A). The variance versus mean current was also analyzed for small open probabilities (5% of the maximum current I), which allowed the single-channel current i to be inferred from a linear approximation: σ_I_^2^ = i**·**I (Fig. 4 B). This method avoided possible contamination due to slight variations in the rising phase of the current, which gave rise to the nonsmooth appearance of the parabola; however, it did not yield a value for the number of channels. Linear fits, for which the ratio σ_Ι_^2^ to I is approximately equal to the single-channel currents, gave values of −3.4 ± 0.4 pA at the apex and −3.5 ± 0.2 pA at the base; no significant difference existed for this method between the locations (two-tailed *t* test P = 0.5).

Fig. 4 C shows the distribution of single-channel currents inferred from parabolic fits to variance versus mean current at the two cochlear locations, with means of −3.6 ± 0.4 pA (*n* = 10) at the apex and −3.7 ± 0.4 pA (*n* = 8) at the base, the two values not being significantly different (two-tailed *t *test P = 0.55). A range of maximum current amplitudes at each location was obtained by recording at different postnatal ages from P2 onward, over which period the maximum current increased in amplitude to reach a saturating level at P7 (Beurg et al., 2018). The number of channels per bundle N_MET_ inferred from the noise analysis was proportional to the maximum current and was larger for basal than apical OHCs (Fig. 4 D). A fit to all points gave an inverse slope (pA / channel) of −3.7 pA (*n* = 20) at −84 mV holding potential, equivalent to a unitary conductance of 44 pS. One interpretation of this analysis is that the tonotopic gradient in OHC channel size (Fettiplace and Kim, 2014) may be produced by a gradient in channel number rather than channel conductance (Ricci and Fettiplace, 1997; Beurg et al., 2018).

Recordings from IHCs using noise analysis gave channel parameters comparable to those for OHCs (Fig. 4 D), with a mean single-channel current of −3.6 ± 0.3 pA (*n* = 5). In comparison, single-channel events after BAPTA treatment had an amplitude of −6.2 pA (Beurg et al., 2018). Previous MET channel measurements based on noise analysis gave single-channel currents in IHCs as −13 pA (Fig. 4 F; see Pan et al., 2018) compared with our values of −3.5 pA (at a similar −80 mV holding potential). The reasons for the discrepancy are unclear.

### MET channel conductance in a *Tmc1* mutant

**Figure 5.**
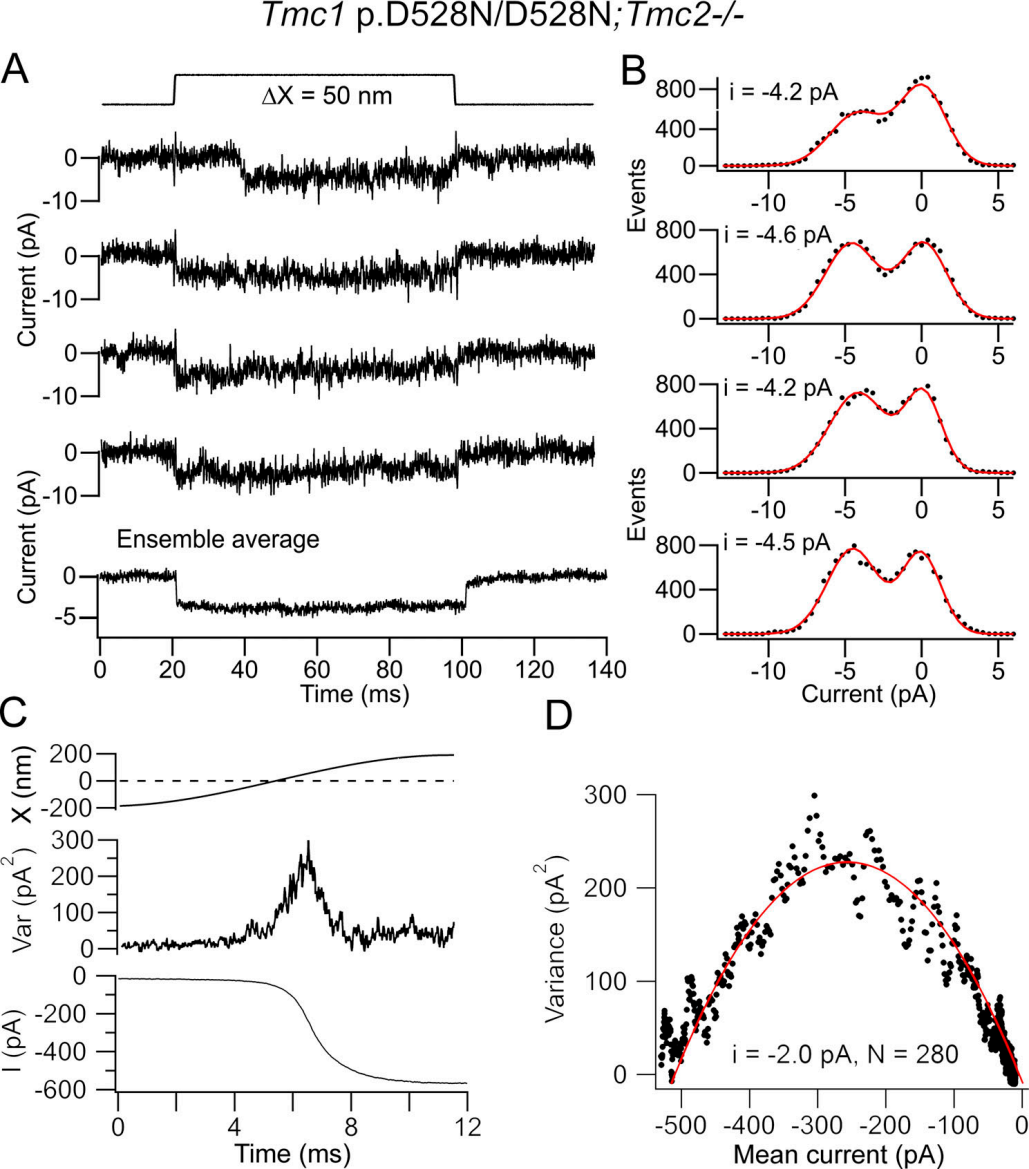
**Single channels in apical OHCs of *Tmc1* p.D528N/D528N; *Tmc2^−/−^* P5 mice. (A)** Four single-channel events for bundle deflection (top) with ensemble average of 35 presentations (bottom). **(B)** Amplitude histograms of the recordings in A, with two Gaussian fits giving peak currents of −4.2 to −4.6 pA. **(C)** Response to a half cycle of bundle deflection showing the mean current I (bottom) and current variance (top). **(D)** Variance plotted against mean current, with smooth red line fit with Eq. 1 (i = −2.0 pA and N_MET_ = 280 channels). Channel events and noise analysis from different OHCs. Holding potential, −84 mV. Var, variance.

To test whether the discrepancy between the two methods still held if the channel conductance was altered, we engineered a missense mutation, *Tmc1* p.D528N, which substantially reduced the single-channel conductance (Beurg et al., 2021). Homozygous *Tmc1* p.D528N mutants were deaf by P28. According to recent modeling, the D528 site is thought to be in transmembrane domain 6 of TMC1 near the extracellular face of the hypothetical pore region (Ballesteros et al., 2018). MET channel conductance was assayed using both techniques. Examples of single-channel events showed amplitudes of −4.2 to −4.5 pA (Fig. 5, A and B). Recordings in five apical OHCs gave a mean amplitude of −4.5 ± 0.3 pA. When noise analysis was applied, a single-channel current of −2.0 pA was inferred (Fig. 5, C and D). Collected noise measurements gave a mean single-channel current for *Tmc1* p.D528N/D528N; *Tmc2^−/−^* of −2.4 ± 0.1 pA (*n* = 5), which was about a third smaller than from *Tmc1^+/+^; Tmc2^−/−^* (significantly different, two-tailed *t *test P = 0.001). As with the *Tmc1^+/+^; Tmc2^−/−^* channels, the single-channel current derived from current noise analysis was about half the size of the unitary current events measured in the *Tmc1* p.D528N mutant (ratio of noise to events = 0.53).

### Modeling of noise analysis

**Figure 6.**
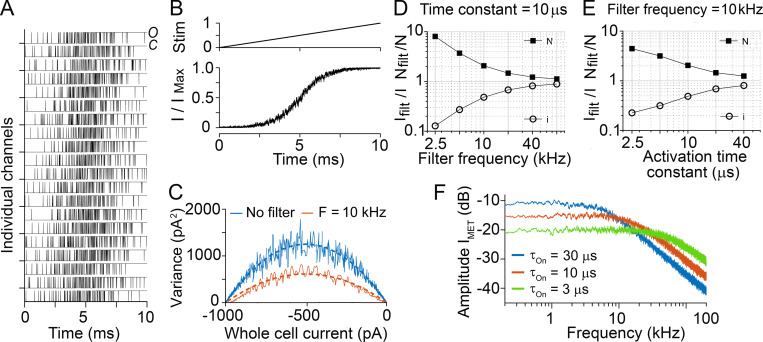
**Simulations of stochastic gating of two-state channel.** **(A)** Stochastic activity in 20 of the 200 channels simulated during a ramp stimulus that activated the channels from closed (*C*) to open (*O*). **(B)** Stimulus (top) to produce the current I scaled to I_Max_, shown below for 100 repetitions. **(C)** Current variance plotted against mean current in the absence of an output filter (blue, i = −10 pA) and after low-pass filtering at 10 kHz (red, i = −4.9 pA) for a channel activation time constant of 10 μs. **(D)** Changes in the apparent values of the single-channel current (i, open circles) and the number of channels (N_MET,_ filled squares) as a function of the low-pass filter frequency. Activation time constant of channel fixed at 10 μs. **(E)** Changes in the apparent values of the single-channel current and the number of channels as a function of channel activation time constant with low-pass filter frequency fixed at 10 kHz. **(F)** Amplitude spectra of simulated MET current for channels with different activation time constants. Three cases were simulated with channel activation time constants τ_ON_ of 3 μs, 10 μs, and 30 μs at a resting open probability of 0.1. The frequency spectra show that the channel behaves like a first-order filter with half-power frequencies (= 1/2π τ_ON_) of 53 kHz (green), 15.9 kHz (orange), and 5.3 kHz (blue). const, constant; filt, filter; Stim, stimulus.

The channel conductance derived from noise analysis may be underestimated if the channel gating is fast compared with the output filtering of the current (Heinemann and Conti, 1992; Alvarez et al., 2002). For the MET channel in mammalian OHCs, the activation time constant of the MET channel is unknown but must be very fast, in the microsecond range (Ricci et al., 2005; Doll et al., 2012), to encode sounds in the ultrasonic hearing limit of rodents. We estimated the limitation imposed by the recording bandwidth by simulating 200 MET channels modeled with two-state (closed to open) channel kinetics. Channel kinetic parameters were chosen to result in a 10-μs activation time constant at a channel open probability of 0.1. Examples of stochastic channel activity are shown in Fig. 6 A, with the channel activated over a 10-ms period evoking an increase in open probability from 0 to 1 (Fig. 6 B). The process was repeated 100 times and analyzed as for the experimental data to generate the variance–current plots (Fig. 6 C). In the absence of filtering, the variance was larger than when a 10-kHz low-pass filter was imposed on the output, agreeing with existing theory (Heinemann and Conti, 1992), the fits giving channel currents of 10 pA (raw unfiltered) and 4.9 pA (filtered) at 10 kHz.

To illustrate the interplay between the filter frequency and the channel noise, two sets of plots were obtained. First, the generated dataset was low-pass filtered at different frequencies using an 8-pole filter with corner frequencies from 2.5 to 80 kHz, and the “apparent” values of single-channel current and numbers of channels were determined (Fig. 6 D). In the other case, a channel with different activation time constants between 2.5 and 40 μs was simulated (Fig. 6 E). Each dataset was then low-pass filtered at 10 kHz, as done with the experimentally recorded MET currents, before deriving the apparent values of single-channel current and numbers of channels. The results of the simulations imply that for channels with fast activation rates, the variance, and hence unitary current, will be significantly underestimated. For the simulations plotted, no channel adaptation was incorporated. However, when adaptation with a time constant of 0.2 ms (Kennedy et al., 2003) was added, the apparent single-channel conductance was identical to that without adaptation. Comparing the simulation with the experimentally derived values provided a way of estimating the channel kinetics. The experimental data on the apical OHCs gave a ratio of channel currents from noise analysis to single-channel events of 0.49. Assuming that the discrepancy reflects an underestimate of the noise-derived value because of limited recording bandwidth, such an error would occur for a channel activation time constant of 10 μs (Fig. 6 E) when the responses were low-pass filtered at 10 kHz. The activation step of the two-state channel in the model behaves like a first-order low-pass filter. This was demonstrated by constructing amplitude spectra of simulated MET currents for channels with different activation time constants (Fig. 6 F), calculated at a resting open probability of 0.1. The frequency spectra for channel time constants of 3 μs, 10 μs, and 30 μs had half-power frequencies of 53 kHz, 15.9 kHz, and 5.3 kHz, respectively, exemplifying the frequency range achievable.

**Figure 7.**
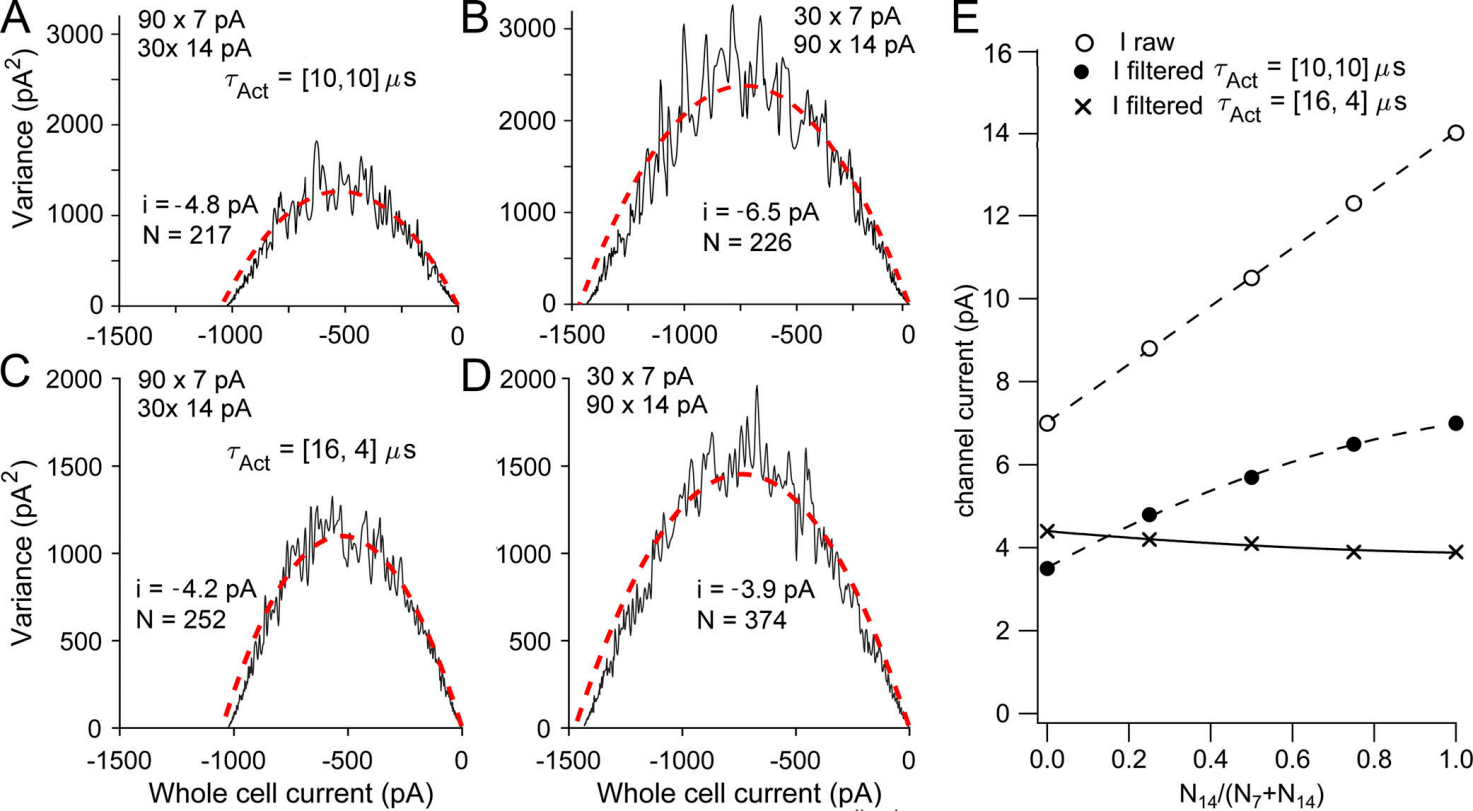
**Simulations of stochastic gating of 120 channels of two different single-channel currents mixed in different proportions. (A)** Variance–mean plot for mixing 90 7-pA and 30 14-pA channels, fit to Eq. 1, gave i = −4.8 pA. **(B)** Variance–mean plot for mixing of 30 7-pA and 90 14-pA channels, fit to Eq. 1, gave i = −6.5 pA. In A and B, both 7-pA and 14­-pA channels have the same activation time constants τ_Act_ = 10 μs. **(C)** Variance–mean plot for mixing 90 7-pA and 30 14-pA channels, fit to Eq. 1, gave i = −4.2 pA. **(D)** Variance–mean plot for mixing of 30 7-pA and 90 14-pA channels, fit to Eq. 1, gave i = −3.9 pA. In C and D, 7-pA and 14-pA channels have activation time constants τ_Act_ = 16 μs and 4 μs, respectively. In all conditions, both channel currents were filtered at 10 kHz. **(E)** Plot of apparent channel current versus proportion that were 14-pA channels [N_14_ / (N_7_ + N_14_)]. Currents unfiltered (raw, open circles), channels with identical kinetics (τ_Act_ = 10 μs), filtered at 10 kHz (filled circles), and channels with different kinetics (7-pA channel τ_Act_ = 16 μs; 14-pA channel τ_Act_ = 4 μs) all filtered at 10 kHz (crosses). Note that with mixing of 7-pA slow channels and 14-pA fast channels, the apparent conductance is virtually unchanged.

We also simulated combinations of MET channels of two different amplitudes in different ratios. We used 7 pA and 14 pA, the number of channels together amounting to a total of 120, and initially assumed that both channel types had the same 10-μs activation time constant. 7 pA and 14 pA were selected as mimicking apical and basal OHC channels, respectively. If all the channels were 7 pA, filtering at 10 kHz would give an estimate from noise analysis of 3.4 pA, and if all channels were 14 pA, noise analysis would yield a 6.9-pA channel. Mixing 90 7-pA and 30 14-pA channels still gave a good noise variance–mean plot that could be fit with a single parabola (Eq. 1), yielding an apparent amplitude of 4.8 pA (Fig. 7 A). Mixing 30 7-pA channels and 90 14-pA channels gave an apparent amplitude of 6.5 pA (Fig. 7 B). An important conclusion from these simulations is that if channels of two amplitudes exist, whatever their relative proportions, only a single peak occurs in the semicircular noise plots, but the apparent single-channel current varies with the relative proportions of the two channel sizes (Fig. 7 E, filled circles). However, instead of channels with identical kinetics, the mixing of small slow channels with large fast channels gave a different result. We assumed that the activation time constant of the small (7 pA) channel was 16 μs and the activation time constant of the large (14 pA) channel was 4 μs. These values were chosen to give a fourfold difference with a mean of 10 μs. With differences in both channel size and kinetics, there was surprisingly little change in the apparent channel current (Fig. 7, C–E), the contribution of the faster channel being filtered to a greater extent. This lack of a gradient in the noise-derived channel size agrees with the experimental results (Fig. 4 C). A tonotopic gradient in both activation and adaptation kinetics has been previously reported for turtle auditory hair cells (Ricci, 2002; Ricci et al., 2005), although direct measurements of mammalian activation time constants have not yet been made.

### Effects of filtering on the noise-derived channel conductance

**Figure 8.**
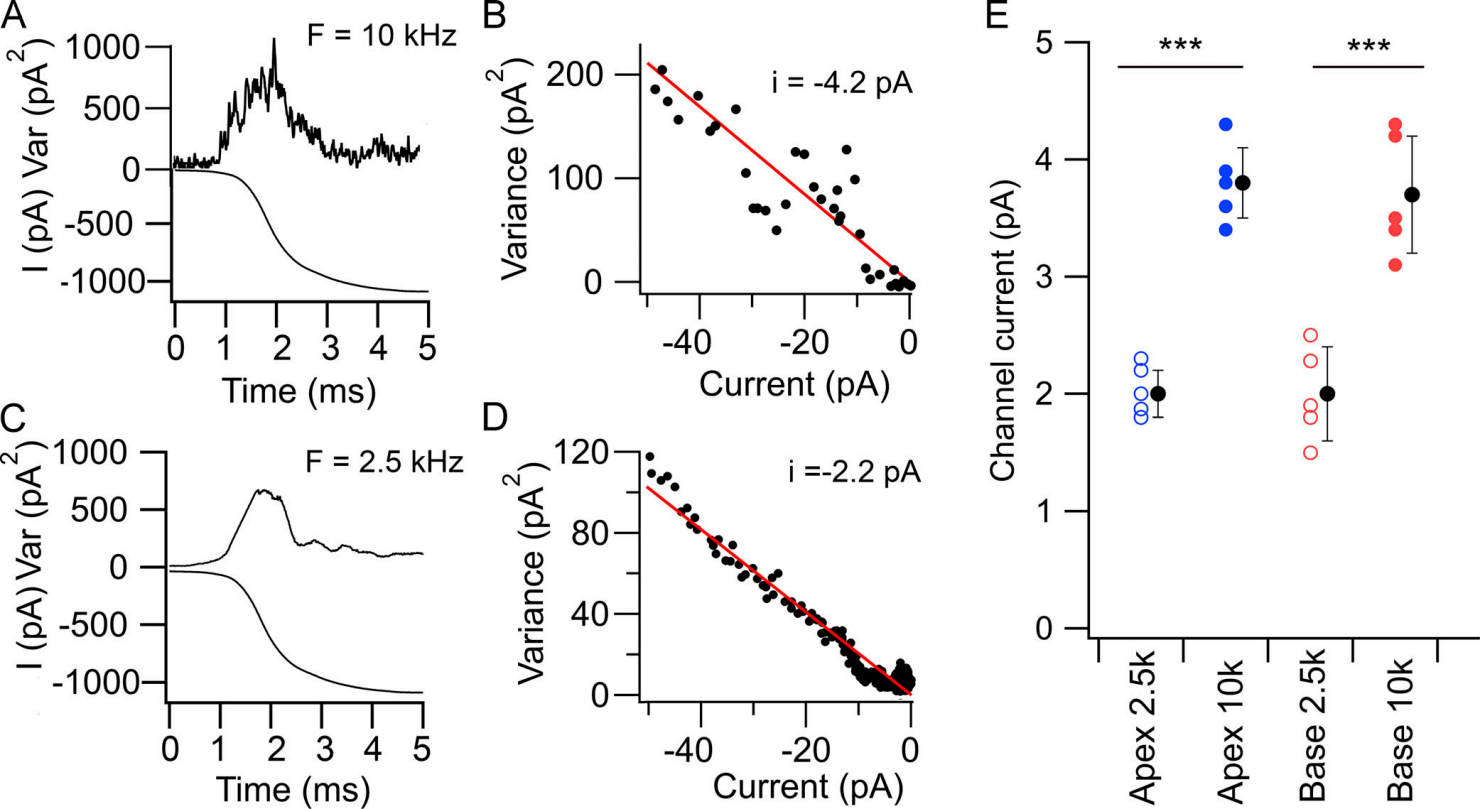
**Effects of filtering on noise-derived MET single-channel currents. (A)** Response to a half cycle of bundle deflection recorded with a 10-kHz low-pass filter showing the current variance (top) and the mean current (bottom) for 45 presentations. **(B)** Plot of variance against mean current for small stimuli; linear fit gave i = −4.2 pA. **(C)** Response to a half cycle of bundle deflection recorded with a 2.5-kHz low-pass filter for the same cell as A showing the current variance (top) and the mean current (bottom) for 45 presentations. **(D)** Plot of variance against mean current for records in C; linear fit gave i = −2.2 pA. Note that the 2.5-kHz filter smoothed the variance and reduced the apparent unitary conductance. All measurements on a P5 mouse. **(E)** Collected results of apparent channel current for five apical OHCs filtered at 10 kHz (filled blue circles) and 2.5 kHz (open blue circles) and for five basal OHCs filtered at 10 kHz (filled red circles) and 2.5 kHz (open red circles); filled black circles give mean ± SD. ***, P = 0.0006 by paired *t* test. There was no significant difference between the means for apical and basal results at 2.5 or 10 kHz. In all panels, holding potential, −84 mV. Var, variance.

In light of the modeling, we tested the notion that the smaller apparent single-channel current is attributable to output filtering by recording MET currents from OHCs under two different low-pass filtering conditions, one at 10 kHz and the other at 2.5 kHz. Noise analysis indicated that reducing the frequency of the output filter reduced the apparent unitary current. Fig. 8, A and B, shows the results in an OHC where the current was filtered with an 8-pole low-pass filter at 10 kHz, which was the usual experimental condition; the unitary currents were −4.2 pA from the small stimulus linear fit (Fig. 8 B). If the MET current in the same cell were filtered at 2.5 kHz, the variance trace was smoother (Fig. 8 C) and the unitary current smaller at −2.2 pA (Fig. 8 D). For this cell, the ratio of current values at 2.5 and 10 kHz was 0.52. Recordings at pairs of filter frequencies were obtained in five apical and five basal OHCs; the ratio of single-channel currents at 2.5 and 10 kHz was similar at the two locations (0.52 ± 0.05 in apical cells and 0.53 ± 0.04 in basal cells; Fig. 8 E). These results confirm that filtering of the currents causes a significant error in the inferred unitary MET conductance, but we could detect no significant difference between the locations (two-tailed *t *test P = 0.36). While this experimental observation qualitatively confirms the modeling results, there is a quantitative discrepancy because using the results of the simulation, the ratio of channel currents at 2.5 kHz and at 10 kHz would be 0.27. The reason for this discrepancy is unknown, but it cannot be attributed to limitation imposed by the recording system. The mean recording time constant determined from the series resistance and cell capacitance for the 10 recordings reported above was 17 ± 3 μs, corresponding to a half-power frequency for a single-pole filter of 9.2 ± 1.5 kHz.

## Discussion

**Figure 9.**
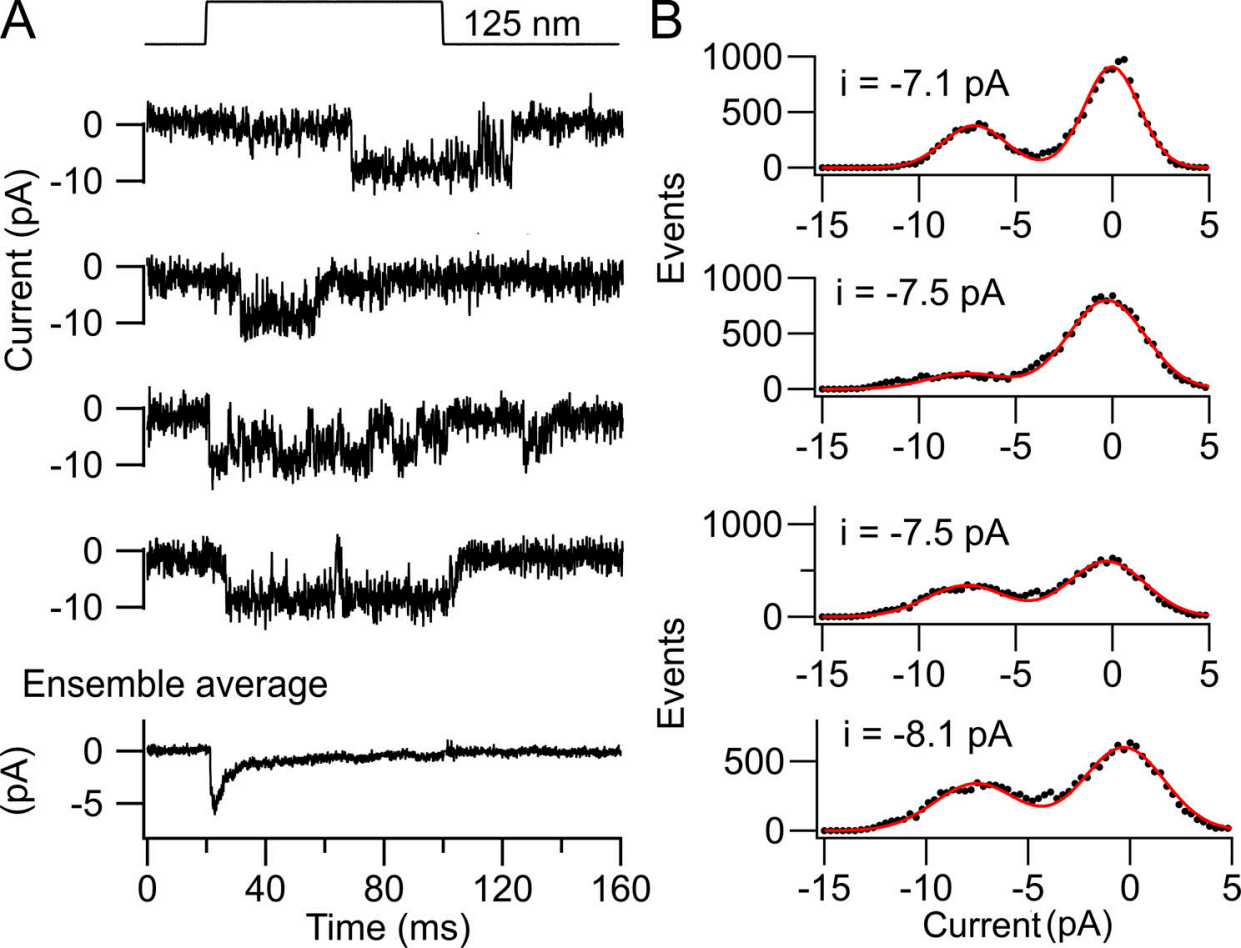
**MET channels recorded in an apical OHC from a P2 *Tmc1^+/+^; Tmc2^−/−^* neonatal mouse without use of BAPTA. (A)** Four single-channel traces and an ensemble average in response to bundle deflection shown at the top. **(B)** Amplitude histograms of the traces in A. The mean channel amplitude was −7.4 ± 0.6 pA from 49 traces. Holding potential, −84 mV.

The main conclusion of this work is that applying noise analysis to MET currents in apical OHCs predicts a single-channel current of about half the size of that derived from directly observing single-channel events following destruction of most of the tip links by BAPTA treatment. It might be argued that the BAPTA exposure has somehow affected channel structure so as to alter its conductance. However, this seems unlikely since BAPTA saline was applied only briefly before the Ca^2+^ in the apical solution was returned to normal at 1.5 mM. Furthermore, MET channels of comparable size can occasionally be recorded without BAPTA treatment in OHCs, with minimal macroscopic currents, early in development (Fig. 9). Recordings from two apical OHCs from P2 *Tmc1^+/+^; Tmc2^−/−^* mice gave a mean conductance of 88 ± 13 pS, comparable to the BAPTA value 85 ± 3 pS at the same cochlear location. Similar values for MET conductance values (112 ± 13 pS, *n* = 3 cells) have previously been reported from spontaneous events in mouse apical OHCs (Géléoc et al., 1997). The smaller conductance value derived from noise analysis may arise from the current variance being underestimated because of filtering of rapid current transients as the channel switches between open and closed states (Heinemann and Conti, 1992; Alvarez et al., 2002). We examined the limitations imposed by filtering by simulating channel gating with a variable low-pass output filter (Fig. 6) and by changing the filter cutoff experimentally (Fig. 8). The modeling indicated that if the MET channel had an activation time constant of 10 μs, noise analysis with output filtering of 10 kHz would cause a twofold underestimate of the channel conductance, as observed experimentally. We therefore propose that a 10-μs value for the time constant may approximate the kinetics for low-level activation of the apical OHC MET channel. This time constant was inferred from our experimental measurements of the MET current at room temperature (∼22°C). As such, this activation rate is faster than any other known channel (Hille, 2001), but it will be even faster at mouse body temperature. Assuming a Q_10_ of 2.1 for the temperature dependence of MET current kinetics (Corey and Hudspeth, 1983; Crawford et al., 1989), the OHC MET time constant extrapolated to 37°C is ∼3 μs, which is equivalent to a half-power frequency of 53 kHz. Amplitude spectra for simulated records of MET channels show that the channel behaves like a first-order filter with half-power frequency (1/2π.τ_ON_), depending on the channel activation time constant τ_ON_ (Fig. 6 F). A cutoff frequency of 53 kHz is sufficiently high to enable faithful encoding of sounds over most of the mouse auditory range that extends up to 70 kHz (Taberner and Liberman, 2005).

The noise analysis predicted similar channel amplitudes in basal and in apical OHCs (Fig. 4 C) despite single-channel events at the base being apparently twice as large as those at the apex; for basal OHCs, the ratio of single-channel currents from noise analysis and channel events was 0.29. If the same filtering argument pertains for apical OHCs, larger channel events in basal OHCs would be expected to have a faster activation time constant than those at the apex. Modeling the mixing of small (7-pA) and large (14-pA) channels indicated that the noise-derived current depended on the fraction of large channels present (Fig. 7 E), ranging from 3.5 to 6.9 pA depending on the proportion of large channels present. However, if the two channel types also had different kinetics, noise analysis would minimize the difference in the noise-derived values (Fig. 7 E). Tonotopic differences in the time constant of fast adaptation has been previously reported for turtle and mammalian auditory hair cells (Ricci et al., 2005), although the mammalian activation time constant has not yet been measured. If the time constant in basal OHCs is 4 μs (at room temperature) as used here for modeling, this predicts an even higher upper frequency limit >100 kHz at 37°C, adequate to encompass the entire auditory range of all mammals.

If there were no tonotopic differences in activation kinetics between apex and base, an alternative hypothesis is that the apical and basal MET channels are identical in kinetics and size (∼7 pA), but there are more channels at the transduction site in each stereocilium of a basal OHC than an apical one (Beurg et al., 2018). This would involve multiple channels from one stereocilium being simultaneously activated and summing to produce a larger apparent conductance. Methods with faster recording speeds may be needed to fully address this question.
